# Well-controlled gestational diabetes mellitus without pharmacologic therapy decelerates weight gain in infancy

**DOI:** 10.3389/fendo.2022.1063989

**Published:** 2022-12-19

**Authors:** Chao Li, Yixi Cai, Yinying Li, Bin Peng, Yongfang Liu, Zhenming Wang, Ting Yang, Yirong Hu, Yajun Fu, Tingmei Shi, Hong Peng, Yue Zhang, Jie Chen, Tingyu Li, Li Chen

**Affiliations:** ^1^ Ministry of Education Key Laboratory of Child Development and Disorders, Department of Growth, Development, and Mental Health of Children and Adolescence Center, National Clinical Research Center for Child Health and Disorders, China International Science and Technology Cooperation Base of Child Development and Critical Disorders, Children's Hospital of Chongqing Medical University, Chongqing, China; Chongqing Key Laboratory of Child Health and Nutrition, Chongqing, China; ^2^ Chongqing Key Laboratory of Child Health and Nutrition, Chongqing, China; ^3^ Department of Child Health Care, The First People's Hospital of Chongqing Liangjiang New Area, Chongqing, China; ^4^ School of Public Health and Management, Department of Health Statistics, Chongqing Medical University, Chongqing, China

**Keywords:** gestational diabetes mellitus, growth, offspring, lifestyle management, glucose control

## Abstract

**Aim:**

There are no prospective longitudinal studies on the association between well-controlled gestational diabetes mellitus (GDM) without pharmacologic therapy and the physical growth of offspring in infancy. We aimed to identify the trajectories in physical growth (from 0–12 months of age) in the offspring of mothers with well-controlled GDM without pharmacologic therapy in a prospective cohort in China.

**Methods:**

This study included 236 offspring of mothers with GDM and 369 offspring of mothers without GDM. Mothers with GDM were not on pharmacologic therapy. The length and weight of infants were measured at 0, 1, 3, 6, and 12 months. Linear mixed-effect models and linear mixed-effect models were applied.

**Results:**

The fully adjusted model showed that the weight-for-age z-score (WAZ), length-for-age z-score (LAZ), and BMI-for-age z-score (BMIZ) were similar at birth for the GDM and control groups. However, subsequent increases in WAZ and BMIZ for the GDM group lagged the increases for the control group at the subsequent periods of observation, 0–1, 0–6, and 0–12 months.

**Conclusions:**

Well-controlled GDM without pharmacologic therapy may normalize physical growth of offspring at birth and decelerate their weight gain in infancy. Whether glycemic control can mitigate the long-term effects of GDM on the growth trajectory in offspring remains unclear.

## 1 Introduction

Gestational diabetes mellitus (GDM) is defined as diabetes diagnosed in the second or third trimester of pregnancy that was not clearly overt diabetes prior to gestation (1). Globally, approximately one in six infants are exposed to hyperglycemia *in utero*, with 14% being born to mothers with GDM (2). After adopting new diagnostic criteria for GDM (outlined by the International Association of Diabetes and Pregnancy Study Group (2010), IADPSG2010), the prevalence of GDM was 12.8%–16.7% due to regional differences in mainland China (3). More and more women are becoming overweight and obese because of lifestyle changes, such as sedentary lifestyle, lack of exercise, and changes in eating habits. Therefore, the prevalence of GDM is expected to continue to increase.

GDM may affect the short- and long-term health of the mother, including an increased risk for hypertensive disorders of pregnancy and cesarean section delivery, as well as an increased risk of developing type 2 diabetes in the future (4). However, the relationship between GDM and offspring health has attracted the most attention. GDM may have a significant “cross-generational effect,” with offspring exposed to GDM being at the risk for adverse outcomes such as macrosomia, fetal hypoglycemia, cardiometabolic disorders, and type 2 diabetes (4). These adverse offspring outcomes may be due to an abnormal intrauterine environment triggered by maternal hyperglycemia (5). In recent years, the short-term outcomes of offspring of mothers with GDM (OGDM) have been improved by anti-glycemic therapy, with a decrease in the incidence of macrosomia and large for gestational age (LGA) has been reduced (6, 7). With regards to GDM management, pharmacologic therapy is required in only 17-30% of cases (8–10). Lifestyle management is one of the most important intervention for GDM management and is prioritized. Yet, there is little research available on whether well-controlled GDM without pharmacologic therapy can reduce fetal overgrowth to provide appropriate guidance for the management of pregnant women with GDM.

Sidell et al. noted that offspring exposed to GDM without pharmacologic therapy had a lower body mass index (BMI), compared to offspring not exposed to GDM as controls, at 6–24 months of age. However, the relationship between the BMI of offspring in early life, from 0 to 6 months of age, and maternal glycemic control in pregnancy has not been investigated (11). A previous study did report that the BMI in OGDM increased slowly at 0–6 months of age, compared to a control group of offspring not exposed to GDM, but with a rapid increase at 48-72 months of age. However, this study combined mothers with mild GDM, GDM, medicated GDM, and unmedicated GDM. As well, maternal glycemic control in pregnancy was not mentioned in the study (12).

Abnormal intrauterine environments during pregnancy have a profound impact on the growth of children of all ages, as well as on adult health (13). Therefore, it is necessary to conduct longitudinal studies with mother and child cohorts to provide stronger information for offspring intervention targets. Accordingly, our aim in this study was to clarify the effects of well-controlled GDM, without pharmacologic therapy, on the physical growth of offspring at birth and on their growth trajectories in the first year of life, adjusting for pre-pregnancy maternal BMI, gestational weight gain, and other confounders. Findings of our study may provide powerful theoretical guidance for rapid response to GDM and, thus, has public health and clinical significance.

## 2 Material and methods

### 2.1 Study population and design

The study was approved by the institutional review board of our institution. The study protocol has been published (14). This prospective cohort study using data was based on the birth cohort data from a secondary hospital in Chongqing, China. The study sample included the offspring of mothers with well-controlled GDM without pharmacologic therapy (GDM group) and the offspring of mothers without GDM (control group), recruited at birth between June 2019 and June 2020. Within 48 hours of delivery, mothers and their offspring who volunteered to participate in this study were included in the GDM and control groups according to the inclusion and exclusion criteria and gave written informed consent. Mothers completed a questionnaire, within 48 h of delivery, to record the following information: maternal height, pre-pregnancy weight reported at the first prenatal assessment at about 8 weeks of gestation, prenatal weight, level of education, average monthly household income, and mode of delivery; and offspring sex, gestational age, date of birth, birth weight, recumbent birth length, and Apgar score. Post-natal assessments of the offspring were conducted at post-natal months 1, 3, 6, and 12, and included recumbent length and weight as anthropometric data and disease status; the feeding pattern from post-natal 0-6 months was also recorded.

### 2.2 Inclusion and exclusion criteria

All pregnant women who regularly visited the outpatient obstetrics department were offered a 75-g 2-h oral glucose tolerance test (OGTT) at 24–28 weeks of gestation. The inclusion criteria were as follows: term delivery, singleton fetus, and no history of perinatal asphyxia or serious diseases affecting offspring growth and development. All pregnant women who regularly visited the outpatient obstetrics department were offered a 75-g 2-h oral glucose tolerance test (OGTT) at 24–28 weeks of gestation. The exclusion criteria were as follows: mothers without OGTT results; mothers who have received drugs interfering with glucose homeostasis before and during pregnancy; mothers age > 35 years; mothers with diseases, such as severe systemic disease, pre-pregnancy diabetes, severe infectious diseases, hypertension in pregnancy, intrahepatic cholestasis of pregnancy, severe anemia in pregnancy; and offspring with diseases affecting metabolism and growth.

### 2.3 Blood glucose monitoring in mothers with GDM

GDM was diagnosed according to the 75-g 2-h OGTT at 24–28 weeks of gestation (IADPSG2010 criteria): blood glucose ≥5.1 mmol/L (fasting) or ≥10.0 mmol/L (60 min) or ≥8.5 mmol/L (120 min) (15). Pregnant women with GDM who received lifestyle management without pharmacologic therapy received individualized meal plans and instructions for physical activity and self-monitoring of blood glucose. they were asked to perform self-monitoring of blood glucose (SMBG) throughout the day at least once a week (SMBG comprised peripheral fasting blood glucose (FBG) and 2-h postprandial plasma glucose (2hPBG) a total of four times) in accordance with Chinese guidelines (16) and report their blood glucose (BG) values to doctors at each visit. If the women had poor adherence to SMBG, they were required to visit the hospital regularly (once every 2–4 weeks; i.e., intermittent SMBG) to have their FBG and 2hPBG monitored. We obtained HbA1c values from medical records, and good glycemic control was defined as a blood glucose value < 20% of the recommended target (fasting BG ≤5.3 mmol/L, 2-h postprandial plasma glucose ≤6.7 mmol/L) and HbA1c < 6.5% at each test.

### 2.4 Anthropometric measurement at birth and during infancy

The anthropometric measurement methods have been reported (14). Briefly, the recumbent length and weight of the offspring at birth, 1, 3, 6, and 12 months were measured by nurses following a standardized procedure and using professional examination instruments. We used the world health organization software to calculate the length-for-age z-scores (LAZ), weight-for-age z-scores (WAZ), and BMI-for-age z-scores (BMIZ) at each of the 5 measurement time points.

### 2.5 Statistical analysis

Statistical analyses were conducted using SAS (r) Proprietary Software 9.3 (TS1M0). BMI was calculated as weight divided by the square of height (kg/m^2^) and gestational weight gain as weight on admission for delivery minus pre-pregnancy weight (kg). Continuous variables are presented as the mean ± standard deviation and categorical variables as a count and percentage. Quantitative data between the GDM and control group were compared using a t-test (normally distributed data) or Wilcoxon rank sum test (non-normally distributed data), with qualitative and ordinal data compared using the chi-squared and Wilcoxon rank sum tests, respectively.

After adjusting for confounders, three general linear models were constructed to evaluate the effect of GDM on WAZ, LAZ, and BMIZ at birth. Confounders were adjusted as follows: Model 1: maternal height (m), level of education (secondary, high school, bachelor, or postgraduate level), average monthly household income (CNY <5000, 5,000–9,999, 10,000–14,999, 15,000–19999, and >20,000), gestational age (days), delivery method (cesarean delivery, yes/no), sex (male, yes/no) and feeding patterns (exclusive breastfeeding at 1, 3, and 6 months of age, yes/no); Model 2: Model 1+ pre-pregnancy BMI (kg/m^2^); and Model 3: Model 2+ gestational weight gain (kg).

A repeated-measures analysis of variance (ANOVA) for WAZ, LAZ, and BMIZ was performed. When the data passed Mauchly’s test of sphericity, a two-way ANOVA analysis was used; otherwise, the Greenhouse–Geisser correction was needed. Repeated measurements were described as the mean ± standard error of mean (SEM) and the trajectories of WAZ, LAZ, and BMIZ were plotted. Linear mixed-effect models consider the internal correlation of repeated measurements of variables and consider data with missing values (17). In these models, the Z score was the dependent variable, with group (GMD versus control) and the follow-up time points set as the fixed effects, each offspring as a random effect, and confounding factors (maternal height, level of education, average monthly household income, gestational age, cesarean delivery, sex, pre-pregnancy BMI, gestational weight gain, and feeding patterns) as covariates. The interaction effects (time × group) were included in the model. We used the maximum likelihood estimation to fit the model. The Akaike information criterion was calculated to fit different covariance models, and the UN structure was selected. The associations between GDM and the physical growth trajectories of OGDM were analyzed longitudinally using linear mixed-effect models and controlling for the abovementioned covariables. Analyses were performed with three models. The adjustments of covariates were consistent with those of confounders in the general linear model. The regression coefficients (β estimates) and associated 95% confidence intervals (CI) were described. Statistical significance was set at P < 0.05.

## 3 Results

### 3.1 The descriptive characteristics parameters in two groups

**Figure 1:**
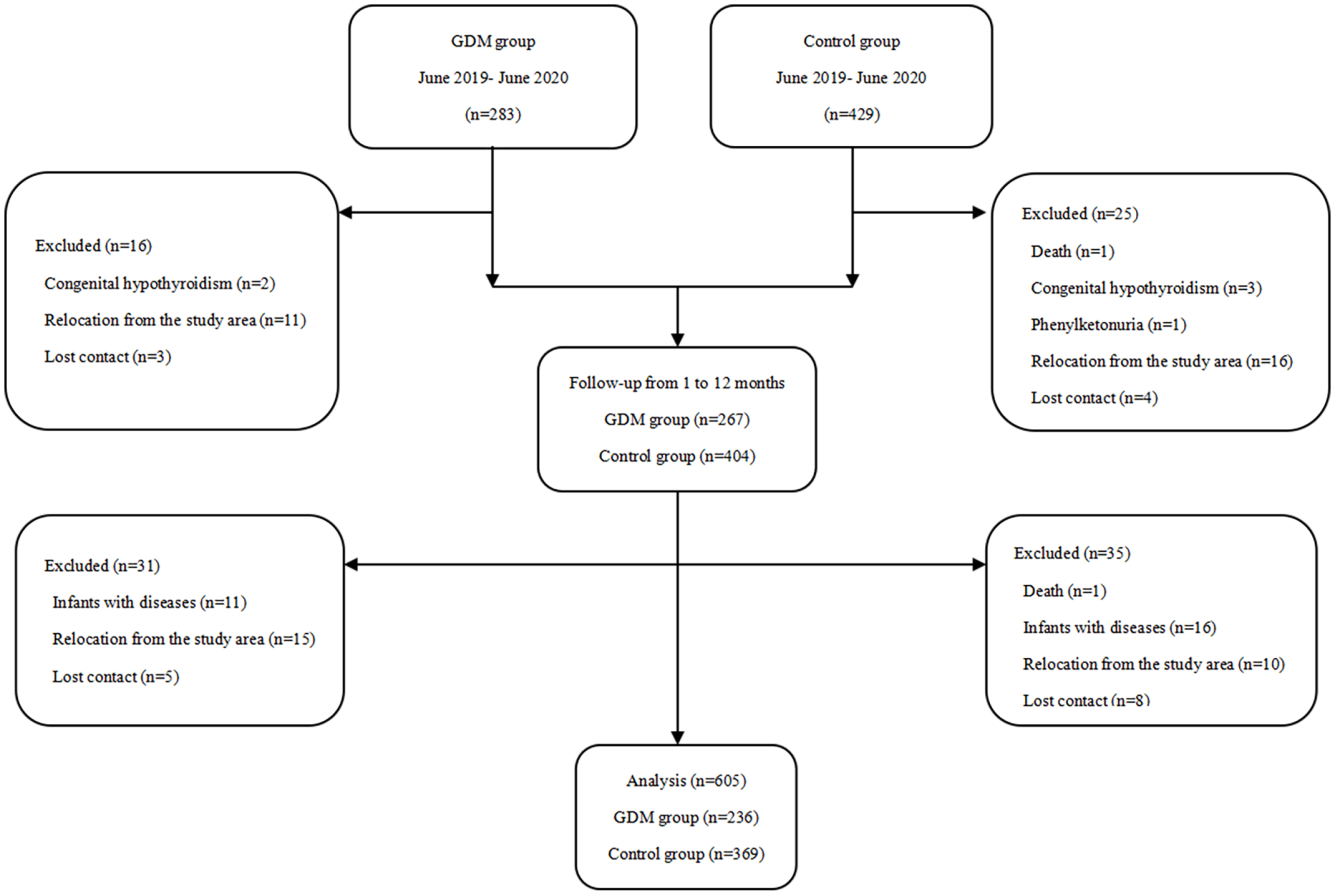
Participant flow chart.

This prospective cohort study included data for 283 offspring of mothers in the GDM group and 429 offspring of mothers in the control group. Of these, 107 infants were excluded due to death (n=2), missing contact information (n=20), diseases affecting metabolism and growth (n=33), and relocation from the study area (n=52). Consequently, 605 offspring (236 in the GDM group and 369 in the control group) were analyzed. The participant flow chart is shown in **Figure 1**. The follow-up rates of 605 offspring at 1-month (32.38 ± 3.76 days), 3 months (95.42 ± 10.20 days),6 months (184.39 ± 11.20 days), and 12 months (373.08 ± 17.14 days) months was 91.6% (554 person-visits), 85.0% (514 person-visits), 80.2% (485 person-visits), and 70.6% (427 person-visits), respectively.

The descriptive characteristics for the GDM and control groups are shown in **Table 1**. Mothers with GDM had a higher rate of cesarean delivery (48.94% vs. 35.16%, *p*<0.001) and higher pre-pregnancy BMI (27.48 ± 3.51 kg/m^2^ vs. 26.93 ± 3.66 kg/m^2^, *p<*0.05) compared with those of the control groups. In contrast, the characteristics of sex, gestational age, feeding patterns, maternal height, gestational weight gain, level of education, and average monthly household income did not different significantly in both groups.

**Table 1:** Descriptive characteristics of the GDM and control groups.

Variables (percentage of missing data for each characteristic)	GDM group (*N*= 236)	Control group (*N*= 369)	*P*
Offspring characteristics			
Male (n, %)	128 (52.24)	190 (51.49)	0.51
Breastfeeding (n, %)			
1 month (8.43%)	143 (63.56)	210 (63.83)	0.95
3 month (15.04%)	160 (74.40)	226 (74.60)	0.87
6 month (19.83%)	131 (68.23)	199 (67.92)	0.94
Gestational age (days) (0.99%)	275.12 ± 5.74	275.95 ± 7.73	0.22
Maternal characteristics			
Height (m) (1.49%)	1.60 ± 0.20	1.58 ± 0.05	0.70
Pre-pregnancy BMI (kg/m^2^) (1.49%)	27.48 ± 3.51	26.93 ± 3.66	0.006
Gestational weight gain (kg) (1.32%)	14.73 ± 4.83	15.37 ± 4.30	0.09
Level of education (n, %)(3.47%)			0.74
Middle school level	39 (16.81)	50 (13.81)	
High school level	57 (24.57)	103 (28.45)	
Bachelor level	134 (57.76)	200 (55.25)	
Postgraduate level	2 (0.86)	9 (2.49)	
Caesarean delivery (n, %) (0.99%)	115 (48.94)	128 (35.16)	<0.001
Average monthly household income, ¥ (CNY) (n, %) (4.46%)			0.93
<5000	29 (12.95)	40 (11.30)	
5000- 9999	109 (48.66)	187 (52.82)	
10000- 14999	64 (28.57)	87 (24.58)	
15000- 19999	15 (6.70)	28 (7.91)	
>20000	7 (3.13)	12 (3.39)	

Values are mean ± SD, or n (%).

GDM, gestational diabetes mellitus; BMI, body mass index.

GDM group: offspring of mothers with well-controlled gestational diabetes mellitus without insulin.

Control group: offspring of mothers without GDM (control group).

Statistical significance was set at P < 0.05.

### 3.2 Glucose data during pregnancy in mothers with GDM

GDM was diagnosed at a mean 24.57 ± 0.94 weeks of gestation. The mean values of fasting glycaemia at OGTT, 1-h glycaemia at OGTT, and 2-h glycaemia at OGTT were 5.25 ± 0.36 mmol/L, 9.22 ± 1.99 mmol/L, and 7.88 ± 1.57 mmol/L, respectively. Target levels of fasting and postprandial blood glucose for *good* glycemic control were achieved in 228 of 236 women (96.6%) in the GDM group, as follows: 4.72 ± 1.41 mmol/L for fasting and 5.63 ± 1.83 mmol/L for postprandial. The mean HbA1c of mothers with GDM in late pregnancy was 4.6 ± 1.7% (30 ± 12mmol/mol).

### 3.3 Anthropometric data of the offspring

**Figure 2:**
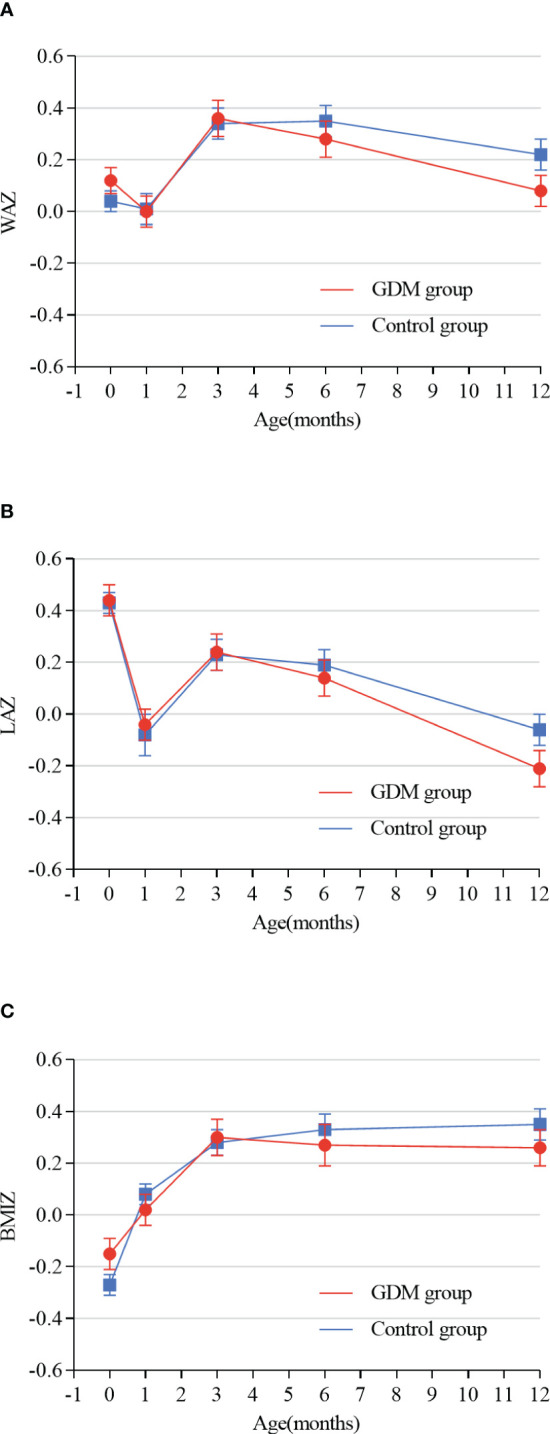
**(A–C)** shows the trajectories of Z-scores from birth to 12 months of age for the GDM and control groups. WAZ weight-for-age z-scores, LAZ: length-for-age z-scores, BMIZ: BMI-for-age z-scores; GDM group: offspring of mothers with well-controlled gestational diabetes mellitus without insulin. Control group: offspring of mothers without GDM.

In the fully adjusted general linear model, WAZ (*p*=0.29), LAZ (*p*=0.74), and BMIZ (*p*=0.25) of OGDM were not different between offspring in the GDM group and control group (**Table 2**). The trajectories of Z-scores for WAZ, LAZ, and BMIZ, using the mean ± SEM values reported in **Table 3**, are plotted for the period of 0-12 months of age in **Figure 2** for both groups. The trajectories for WAZ, LAZ, and BMIZ were not different between the GDM and control group over the period of 0-4 months, with a significant deviation for the GDM group, from the control group, becoming apparent after 4 months of age. As shown in **Figure 2**, the Z-score for all three variables, WAZ, LAZ, and BMIZ, were lower for the GDM than control group at 6 and 12 months of age.

**Table 2:** The general linear model’s parameter outcome comparison of Z scores at birth in both groups.

Outcomes	Model	GDM vs. control group		
		β	SE	*P*
WAZ	Model 1	-0.08	0.06	0.22
	Model 2	-0.06	0.06	0.37
	Model 3	-0.07	0.06	0.29
LAZ	Model 1	-0.03	0.07	0.65
	Model 2	-0.02	0.07	0.78
	Model 3	-0.02	0.07	0.74
BMIZ	Model 1	-0.09	0.07	0.21
	Model 2	-0.07	0.07	0.33
	Model 3	-0.08	0.07	0.25

Data are presented as β and SE.

GDM, gestational diabetes mellitus; WAZ, weight-for-age z-scores; LAZ, length-for-age z-scores; BMIZ, BMI-for-age z-scores.

GDM group: offspring of mothers with well-controlled gestational diabetes mellitus without insulin.

Control group: offspring of mothers without GDM.

Model 1: adjusted for maternal height (m), level of education (secondary, high school, bachelor, or postgraduate level), average monthly household income (CNY, <5000, 5000–9999, 10000–14999, 15000–19999, or >20000), gestational age (days), delivery method (cesarean delivery, yes/no), sex (male, yes/no), and feeding patterns (exclusive breastfeeding at 1, 3, and 6 months of age, yes/no).

Model 2: Model 1+ adjusted for pre-pregnancy BMI (kg/m^2^).

Model 3: Model 2+ adjusted for gestational weight gain (kg).

Statistical significance was set at P < 0.05.

**Table 3:** Outcomes of Z scores in the two groups at different ages ( $\bar{X}\pm SE$ ).

		WAZ	LAZ	BMIZ
0 m	GDM group	0.12 ± 0.05	0.44 ± 0.06	-0.15 ± 0.06
	Control group	0.04 ± 0.04	0.43 ± 0.04	-0.27 ± 0.04
1 m	GDM group	0.00 ± 0.06	-0.04 ± 0.06	0.02 ± 0.06
	Control group	0.01 ± 0.06	-0.08 ± 0.08	0.08 ± 0.04
3 m	GDM group	0.36 ± 0.07	0.24 ± 0.07	0.30 ± 0.07
	Control group	0.34 ± 0.06	0.23 ± 0.06	0.28 ± 0.05
6 m	GDM group	0.28 ± 0.07	0.14 ± 0.07	0.27 ± 0.08
	Control group	0.35 ± 0.06	0.19 ± 0.06	0.33 ± 0.06
12 m	GDM group	0.08 ± 0.06	-0.21 ± 0.07	0.26 ± 0.07
	Control group	0.22 ± 0.06	-0.06 ± 0.06	0.35 ± 0.06

GDM, gestational diabetes mellitus; WAZ, weight-for-age z-scores; LAZ, length-for-age z-scores; BMIZ, BMI-for-age z-scores.

GDM group: offspring of mothers with well-controlled gestational diabetes mellitus without insulin.

Control group: offspring of mothers without GDM.

The results of the linear mixed-effect, fully adjusted, model showed different trajectories of Z-scores for the GDM and control groups (**Table 4**). Compared to the control group, the increases for the GDM group were significantly less than for the control group over the periods of 0–1, 0–6, and 0–12 months, respectively, for WAZ (*p*=0.04, *p*=0.02, and *p*=0.003) and BMIZ (*p*=0.008, *p*=0.04, and *p*=0.01). Of note, the trajectory of the Z-scores for WAZ, LAZ, and BMIZ was not different between the GDM and control group over the 0–3-month period, as was the Z-score trajectory of LAZ over the 0-12 month period. Furthermore, the regression coefficients (βestimates) of the interaction effects (time × group) increased gradually for WAZ and BMIZ at 1, 6, and 12 months of age.

**Table 4:** Linear mixed-effect models of Z-scores.

	Model 1		Model 2		Model 3	
	β(95%CI)	*P*	β(95%CI)	*P*	β(95%CI)	*P*
WAZ						
GDM group	0.11 (-0.02 to 0.24)	0.09	0.09 (-0.03 to 0.22)	0.15	0.10 (-0.03 to 0.23)	0.12
1 m	-0.03 (-0.11 to 0.17)	0.66	0.03 (-0.11 to 0.17)	0.66	0.03 (-0.11 to 0.17)	0.65
3 m	0.35 (0.21 to 0.50)	<0.01	0.36 (0.22 to 0.50)	<0.01	0.36 (0.22 to 0.50)	<0.01
6 m	0.38 (0.23 to 0.53)	<0.01	0.38 (0.24 to 0.53)	<0.01	0.39 (0.24 to 0.53)	<0.01
12 m	0.19 (0.08 to 0.29)	<0.01	0.19 (0.08 to 0.29)	<0.01	0.19 (0.08 to 0.29)	<0.01
GDM group × 1 m	-0.14 (-0.28 to -0.01)	0.04	-0.14 (-0.28 to 0.01)	0.04	-0.15 (-0.28 to -0.01)	0.04
GDM group × 3 m	-0.12 (-0.28 to 0.04)	0.13	-0.13 (-0.28 to 0.03)	0.12	-0.13 (-0.29 to 0.03)	0.12
GDM group × 6 m	-0.20 (-0.37 to -0.03)	0.02	-0.20 (-0.37 to -0.03)	0.02	-0.20 (-0.37 to -0.03)	0.02
GDM group × 12 m	-0.25 (-0.41 to -0.09)	0.00	-0.25 (-0.42 to -0.09)	0.00	-0.25 (-0.42 to -0.09)	0.00
LAZ						
GDM group	0.07 (-0.07 to 0.21)	0.36	0.06 (-0.08 to 0.20)	0.44	0.06 (-0.08 to 0.20)	0.42
1 m	-0.56 (-0.72 to -0.40)	<.01	-0.56 (-0.72 to -0.40)	<.01	-0.56 (-0.72 to -0.40)	<.01
3 m	-0.27 (-0.42 to -0.11)	0.00	-0.27 (-0.42 to -0.11)	0.00	-0.27 (-0.42 to -0.11)	0.00
6 m	-0.30 (-0.45 to -0.14)	0.00	-0.30 (-0.45 to -0.14)	0.00	-0.30 (-0.45 to -0.14)	0.00
12 m	-0.50 (-0.60 to -0.40)	<.01	-0.50 (-0.60 to -0.40)	<.01	-0.50 (-0.60 to -0.40)	<.01
GDM group × 1 m	-0.02 (-0.18 to 0.13)	0.76	-0.02 (-0.18 to 0.13)	0.75	-0.03 (-0.18 to 0.13)	0.75
GDM group × 3 m	-0.02 (-0.18 to 0.13)	0.79	-0.02 (-0.18 to 0.13)	0.77	-0.02 (-0.18 to 0.13)	0.77
GDM group × 6 m	-0.10 (-0.25 to 0.06)	0.22	-0.10 (-0.25 to 0.06)	0.22	-0.10 (-0.25 to 0.06)	0.22
GDM group × 12 m	-0.16 (-0.31 to -0.00)	0.05	-0.15 (-0.31 to 0.00)	0.05	-0.15 (-0.31 to 0.00)	0.05
BMIZ						
GDM group	0.12 (-0.02 to 0.27)	0.10	0.11 (-0.04 to 0.26)	0.14	0.12 (-0.03 to 0.27)	0.12
1 m	0.48 (0.32 to 0.63)	<.01	0.48 (0.32 to 0.63)	<.01	0.48 (0.32 to 0.63)	<.01
3 m	0.69 (0.52 to 0.85)	<.01	0.69 (0.53 to 0.85)	<.01	0.69 (0.53 to 0.85)	<.01
6 m	0.74 (0.57 to 0.91)	<.01	0.75 (0.58 to 0.92)	<.01	0.75 (0.58 to 0.92)	<.01
12 m	0.63 (0.51 to 0.75)	<.01	0.63 (0.50 to 0.75)	<.01	0.63 (0.50 to 0.75)	<.01
GDM group × 1 m	-0.21 (-0.37 to -0.06)	0.01	-0.21 (-0.37 to -0.06)	0.01	-0.21 (-0.37 to -0.06)	0.01
GDM group × 3 m	-0.17 (-0.36 to 0.02)	0.09	-0.17 (-0.36 to 0.02)	0.08	-0.17 (-0.36 to 0.02)	0.08
GDM group × 6 m	-0.21 (-0.41 to -0.01)	0.04	-0.21 (-0.41 to -0.01)	0.04	-0.21 (-0.41 to -0.01)	0.04
GDM group × 12 m	-0.25 (-0.45 to -0.06)	0.01	-0.25 (-0.45 to -0.05)	0.01	-0.25 (-0.45 to -0.05)	0.01

Data are presented as β and 95% CI.

The interaction effects (time × group) were included in the model.

GDM, gestational diabetes mellitus, WAZ: weight-for-age z-scores, LAZ: length-for-age z-scores; BMIZ, BMI-for-age z-scores; CI, confidence interval.

GDM group: offspring of mothers with well-controlled gestational diabetes mellitus without insulin.

Control group: offspring of mothers without GDM.

Model 1: adjusted for maternal height (m), level of education (secondary, high school, bachelor, or postgraduate level), average monthly household income (CNY, <5000, 5000- 9999, 10000- 14999, 15000- 19999, >20000), gestational age (days), delivery method (cesarean delivery, yes/no), sex (male, yes/no) and feeding patterns (exclusive breastfeeding at 1, 3, and 6 months of age, yes/no).

Model 2: Model 1+ adjusted for pre-pregnancy BMI (kg/m^2^).

Model 3: Model 2+ adjusted for gestational weight gain (kg).

Statistical significance was set at P < 0.05.

## 4 Conclusions

The novel contribution of our observational study is the longitudinal reporting of an association between well-controlled GDM without pharmacologic therapy and the physical growth of offspring in the first year of life. We found that WAZ, LAZ, and BMIZ of OGDM were not amplified at birth, but that a period of “catch-down” growth followed. The lag became more pronounced with age between the two groups. These observations were independent of pre-pregnancy BMI, gestational weight gain, and other maternal and infant factors. Our findings support an association between well-controlled GDM and a slower rate of physical growth of offspring, at least up to 12 months of age, the end point of the period of observation in our study.

The Similar physical growth measures at birth between the GDM and control group was consistent with those of previous studies (18, 19). Of note, although Ignell et al (18) reported similar birth weight and length between offspring of mothers with GDM and the control group without GDM in a British sample, maternal glycemic control was not described. In their study, Au et al. (19) further reported a body fat (BF) percentage for offspring of mothers with GDM and those without GDM. In both studies, however, maternal interventions included a combination of lifestyle management and lifestyle management + pharmacologic therapy. Moreover, between-group comparisons were not adjusted for pre-pregnancy BMI and gestational weight gain. In contrast, a systematic review reported significantly greater infant adiposity associated with GDM compared to non-GDM (20); however, this review included infants of all pregnant women with diabetes, including those with type 1 and 2 diabetes and GDM. A recent 2018 systematic review identified that dietary modification interventions might reduce the birth weight of offspring (21). However, most of the studies included in this review had small sample sizes (≤100 participants), with few studies having slightly stronger evidence for reported outcomes. Overall, while existing research indicates important findings on the effects of diabetes in pregnancy and birth weight and adiposity, studies on the associations between GDM without pharmacologic therapy and the physical growth of offspring at birth are still lacking. Our study addresses this gap, with findings that well-controlled GDM without pharmacologic therapy, might normalize the physical growth at birth in infants.

At 0–1 month of age, we did identify a deceleration was observed in the increase of WAZ and BMIZ in OGDM compared to those of the control group, which may be a new finding. In their cross-sectional, rather than longitudinal, observational study, Logan et al. reported that weight and length SDS were significantly lower for OGDM than for a non-GDM control groupt at approximately two weeks of age (22). Conversely, Uebel et al. reported a higher fat mass for offspring of obese mothers with GDM being an independent risk factor for offspring obesity (23). However, the sample size of this study was small and only included women with obesity who had GDM. In our study, we identified a deceleration in the increase in WAZ and BMIZ for OGDM over the period of 0-6 months of age, compared to the control group. This is consistent with the findings of Sidell et al. Who reported that the BMI of OGDM was significantly lower than that of offspring of healthy mothers over the period of 0–6 months of age (11). However, Sidell et al. did not include mothers with GDM. Furthermore, our study identified a deceleration in the increase in WAZ and BMIZ over the period of 0-12 months of age for the GDM compared to the control group. Ignell et al. (18) similarly reported that increases in weight and skinfold thickness were significantly lower over the period of 3-12 months of age in OGDM compared to a control group. However, in contrast to our findings, another study reported that the increases in weight and skinfold thickness was significantly greater from 0-3 months of age and the increase in length was significantly less over the 0-12 month age period (24). A more recent, 2022, study reported offspring of mothers treated for GDM gained more weight in infancy compared to the infants born to mothers with gestational impaired glucose tolerance and healthy mothers (25). However, these studies, included mothers with treated and untreated GDM, did not describe glycemic control during gestation, and did not describe lag in postnatal growth trajectory in OGDM. Of note, the growth trajectories of OGDM in early life were similar to those of individuals who experience obesity rebound in childhood and type 2 diabetes in adulthood (26, 27). Therefore, the age at which “catch-down” weight among OGDM becomes most pronounced remains to be clarified. To address this specific issue, we are continuing the follow-up of our sample to determine if and when a “catch-up” period of growth occurs in OGDM with a longer period of observation.

The mechanisms by which offspring normalize weight at birth and subsequently experience periods of “catch-down” growth are unclear. The strict diagnostic criteria for GDM proposed in the IADPSG2010 guideline resulted in more pregnant women with elevated BG values being identified and treated with aggressive glycemic control. The diagnosis and treatment of mild GDM reportedly reduce the risk for macrosomia and LGA (28), and another study found BF was similar at birth between the well-controlled GDM and control groups (19). Although our study population consisted of mothers who had met the diagnostic criteria for GDM, their BG values were closer to those of mild GDM and were more likely to be controlled at normal levels without pharmacologic therapy. Therefore, our results support the perspective that maternal exposure to lower glucose and good glycemic control may normalize the birth weight of their offspring. Furthermore, gestational weight gain is associated with accelerated fetal growth accelerated fetal growth (29). In our study, gestational weight gain, limited by lifestyle management, was similar between the GDM and control group, which reduced the risk of a higher birth weight in offspring overall. It is important to note that antidiabetic medications may alter fetal growth. A systematic review and meta-analysis of 33 studies found that among the offspring of mothers taking insulin, glyburide, and metformin, those of mothers taking glyburide were the heaviest and those of mothers taking metformin were the lightest (30). In addition, a study in the United States reported that the offspring of mothers using insulin and glyburide as antidiabetic medications had higher birth weights compared to offspring of mothers not receiving any antidiabetic medications (31). Metformin and glyburide cross the placenta and may affect the short-term growth of offspring through specific mechanisms (32). Combined with our findings, we conclude that the growth of offspring unexposed to antidiabetic medications may escape the possible potential adverse effects of the drugs.

After adjusting for the most important confounders (maternal BMI and gestational weight gain) and avoiding possible interference by anti-glycemic drugs, we found that good glycemic control during gestation did not completely protect offspring of mothers with GDM from effects of an adverse intrauterine environment during infancy, from 0-12 month. Therefore, other factors related to hyperglycemia during pregnancy, besides abnormal metabolism induced by hyperglycemia, can affect the growth of offspring. Leptin, a protein encoded by obesity genes, is mainly synthesized and secreted by body fat and is proportional to body fat mass. It regulates eating behavior and energy metabolism (33). OGDM reportedly have higher birth weight and cord blood leptin levels than those of the control group (34, 35). Two other studies found that although there was no significant difference in birth weight between the GDM and control groups, cord blood leptin levels were still higher in the GDM group, which may be related to the relative increase in BF of OGDM (36, 37). Therefore, cord blood leptin levels were higher for the GDM group regardless of amelioration of the classic macrosomic phenotype under maternal glycemic control during gestation.

Clinical and animal studies have found that offspring with high leptin levels do not develop the same “leptin resistance” early in life as adults with obesity who have high leptin levels (34, 35, 38, 39). Parker et al. supported that higher cord leptin levels were associated with slower weight gain from 0–6 months (35). Kaar et al. suggested that cord leptin levels were negatively correlated with weight gain in the first year of life (34). Our study found similar growth trajectories for OGDM. Therefore, we hypothesized that the growth trajectories of OGDM may result from feedback regulation developed *in utero*, with high *in utero* leptin levels in particular slowing down the early postnatal weight gain of OGDM by regulating feeding behavior and metabolism. Further studies are needed to confirm the exact associations between cord blood leptin levels and growth trajectories of OGDM who have a normal birth weight.

Few studies have shown that a higher intake of breast milk is associated with slower and less weight gain in OGDM, over the period of 0-12 months, and that breastfeeding is associated with a lower risk of childhood obesity (40), These findings may be related to lower concentrations of ghrelin and adiponectin in breast milk (41). We collected information on exclusive breastfeeding (yes/no) at three time points in our study sample, and they were all similar. Associations between breastfeeding and weight gain in OGDM need to be further explored.

Our study is unique and meaningful. To date, no prospective longitudinal studies have shown the associations between well-controlled GDM without pharmacologic therapy and the physical growth of offspring at 0-12 months of age. Data on blood glucose monitoring have confirmed that most mothers with GDM can achieve good glycemic control without pharmacologic therapy (8–10). Therefore, our study on the growth trajectories of most OGDM (exposed to relatively low levels of hyperglycemia *in utero*) has practical significance. Furthermore, this study considered possible confounders, particularly pre-pregnancy BMI and gestational weight gain, to confirm the independent effect of GDM on the physical growth of offspring.

The limitations of our study need to be acknowledged. First, our study population comes from a secondary regional hospital in developing regions of the country, with relatively low economic family income and maternal education levels. To maximize the reliability and adherence of SMBG, intermittent monitoring was used by some mothers with GDM through regularly scheduled hospital visits. We strictly screened out well-controlled GDM from the SMBG and medical records. However, compared with frequent SMBG, intermittent SMBG might have resulted in the identification of a smaller number of mothers whose glycemic control was lower than expected. The status of glycemic control requires careful interpretation. Second, we did not have data on cord blood leptin levels and body fat of offspring, and we plan to clarify the associations in another study. Finally, the study lacked groups of OGDM of mothers who received pharmacologic therapy for GDM management to investigate whether there were differences in growth trajectories between OGDM who received different therapies and the offspring with mothers without GDM. In our future study, longitudinal follow-up and continuous attention will be paid to clarify the physical growth pattern of OGDM and provide references for clinical intervention strategies for mothers with GDM.

In conclusion, well-controlled GDM without pharmacologic therapy may normalize the physical growth of offspring at birth and decelerate weight gain in infancy. Continued follow-up will help assess the growth trajectories of offspring and whether good glycemic control alleviates the long-term effects of GDM on offspring. This may provide data support for the most accurate medical monitoring and health management for pregnant women with GDM and their offspring.

## Data availability statement

The data that support the findings of this study are available from the corresponding author, upon reasonable request.

## Ethics statement

The studies involving human participants were reviewed and approved by Institutional Review Board, Children’s Hospital of Chongqing Medical University. Written informed consent to participate in this study was provided by the participants’ legal guardian/next of kin.

## Author contributions

CL and LC conceived and designed. LC, YC, YYL, TY, ZW, HP, YZ, YH, TS and YF implemented the study. CL, YC, and YYL acquired and analyzed data. CL and LC drafted the manuscript. JC, YFL, and TL reviewed and revised the manuscript. BP and CL carried out statistical analysis. All authors approved the final version of the manuscript. LC is the guarantor of this work and, as such, had full access to all of the data in the study and takes responsibility for the integrity of the data and the accuracy of the data analysis.
